# Clinicopathological and epigenetic differences between primary neuroendocrine tumors and neuroendocrine metastases in the ovary

**DOI:** 10.1002/2056-4538.70000

**Published:** 2024-11-08

**Authors:** Merijn CF Mulders, Anna Vera D Verschuur, Quido G de Lussanet de la Sablonière, Eva Maria Roes, Christoph Geisenberger, Lodewijk AA Brosens, Wouter W de Herder, Marie‐Louise F van Velthuysen, Johannes Hofland

**Affiliations:** ^1^ ENETS Center of Excellence, Section of Endocrinology, Department of Internal Medicine Erasmus Medical Center Cancer Institute Rotterdam The Netherlands; ^2^ Department of Pathology University Medical Center Utrecht, Utrecht University Utrecht The Netherlands; ^3^ Department of Radiology and Nuclear Medicine Erasmus Medical Center Rotterdam The Netherlands; ^4^ Department of Gynecologic Oncology Erasmus Medical Center Cancer Institute Rotterdam The Netherlands; ^5^ Institute of Pathology Ludwig Maximilian University Munich Germany; ^6^ Department of Pathology Radboud University Medical Center Nijmegen The Netherlands; ^7^ Department of Pathology Erasmus Medical Center Rotterdam The Netherlands

**Keywords:** neuroendocrine tumor, ovary, primary, metastasis, DNA methylation, immunohistochemistry

## Abstract

Currently, the available literature provides insufficient support to differentiate between primary ovarian neuroendocrine tumors (PON) and neuroendocrine ovarian metastases (NOM) in patients. For this reason, patients with a well‐differentiated ovarian neuroendocrine tumor (NET) were identified through electronic patient records and a nationwide search between 1991 and 2023. Clinical characteristics were collected from electronic patient files. This resulted in the inclusion of 71 patients with NOM and 17 patients with PON. Histologic material was stained for Ki67, SSTR2a, CDX2, PAX8, TTF1, SATB2, ISLET1, OTP, PDX1, and ARX. DNA methylation analysis was performed on a subset of cases. All PON were unilateral and nine were found within a teratoma (PON‐T+). A total of 78% of NOM were bilateral, and none were associated with a teratoma. PON without teratomous components (PON‐T−) displayed a similar insular growth pattern and immunohistochemistry as NOM (*p* > 0.05). When compared with PON‐T+, PON‐T− more frequently displayed ISLET1 positivity and were larger, and patients were older at diagnosis (*p* < 0.05). Unsupervised analysis of DNA methylation profiles from tumors of ovarian (*n* = 16), pancreatic (*n* = 22), ileal (*n* = 10), and rectal (*n* = 7) origin revealed that four of five PON‐T− clustered together with NOM and ileal NET, whereas four of five PON‐T+ grouped with rectum NET. In conclusion, unilateral ovarian NET within a teratoma should be treated as a PON. Ovarian NET localizations without teratomous components have a molecular profile analogous to midgut NET metastases. For these patients, a thorough review of imaging should be performed to identify a possible undetected midgut NET and a corresponding follow‐up strategy may be recommended.

## Introduction

Neuroendocrine tumors (NET) are a heterogeneous group of neoplasms with the ability to produce and/or secrete amines and peptide hormones [1]. These tumors derive from neuroendocrine cells and are mainly encountered in the bronchopulmonary and gastrointestinal tracts, with the small intestine or midgut as the leading primary site in Western countries [2]. Ovarian NET localizations are generally considered rare and are subdivided into primary ovarian NET (PON) and neuroendocrine ovarian metastases (NOM). While PON represents about 0.8–3.5% of all NET [3, 4], NOM occur in about 11.2% of women with NET, with their incidence rate increasing to 25% in women with a midgut NET [5]. The carcinoid syndrome (CS), defined by symptoms of chronic diarrhea and/or flushing in the presence of systemic elevated levels of serotonin or its metabolite 5‐hydroxyindolacetic acid (5‐HIAA) [6], is present in 18–33% of patients with PON and in 51–53% of patients with NOM [4, 7, 8, 9].

As NET incidence has increased 3.7‐ to 6.4‐fold over the previous four decades [10, 11, 12], patients with an ovarian NET are also more frequently diagnosed. However, differentiating between PON and NOM in patients proves to be challenging. Currently, decision‐making is based on the presence of teratomatous components and unilateral disease, which are more prevalent but not exclusive to PON [4, 7, 13]. Two studies have tried to differentiate between PON and NOM with the use of immunohistochemistry, failing to find any differences with the exception of a higher Ki‐67 index for NOM [14, 15]. However, both studies combined PON with teratomous components (PON‐T+) and PON without teratomous components (PON‐T−) in their analysis. An additional tumor classification tool is methylation profiling, which is increasingly being used for tissue‐of‐origin identification [16, 17, 18]. The methylation state of a cancer cell is influenced by both normal developmental processes and oncogenic transformation, serving as a “fingerprint” that traces the cell's epigenetic history and lineage. This highly cell‐type‐specific nature of methylation profiles in cancer might make them well‐suited to aid in distinguishing between PON and NOM. Hence, this study aims to discriminate NOM from PON‐T+ and PON‐T− based on clinical characteristics and the analysis of immunohistochemistry and DNA methylation profiles.

## Materials and methods

### Patient selection

Electronic medical records of the Erasmus MC were screened for all patients with ovarian NET that were presented in this ENETS Center of Excellence between 1991 and 2022. In addition, a search through the Dutch nationwide network and registry of histo‐ and cytopathology (PALGA) was performed to identify additional patients and to obtain archived pathological material from other centers throughout The Netherlands [19]. Primary inclusion criteria were either having a histopathologically proven ovarian NET or somatostatin receptor (SSTR) functional imaging suggesting the presence of an ovarian NET. Patients with neuroendocrine carcinomas (NEC), mixed neuroendocrine nonneuroendocrine neoplasms (MiNEN), and those without available clinical data were excluded. For this retrospective cohort study, the need for written informed consent was waived by the medical ethical committee of the Erasmus MC. Additionally, nonovarian samples were included for methylation analysis, which were collected from the University Medical Center Utrecht (UMCU) and Erasmus MC. DNA methylation data were obtained with approval by the UMCU Biobank Research Ethics Committee (TCBio protocol number: 21‐507).

### Outcomes

The primary endpoint of this study was the immunohistochemistry and DNA methylation profile of the ovarian NET and its ability to discriminate among NOM, PON‐T+, and PON‐T−. A PON was defined as an ovarian NET without evidence for a different primary origin. Secondary endpoints were patient characteristics, including age at diagnosis, metastases at diagnosis and at the end of follow‐up, lateralization, overall survival (OS), the presence of CS, defined according to the most recent ENETS guidance paper [6], and the biochemical and subjective symptomatic response to oophorectomy of patients with CS. Biochemical response was defined as a >50% drop in urinary 5‐HIAA in patients with measurements within 1 year of the oophorectomy. OS was determined from the time of ovarian NET diagnosis and was censored if the patient was lost to follow‐up. Patients' follow‐up information was updated until July 2023.

### Histopathologic assessment

Diagnosis and other histopathologic characteristics were revised according to the 2022 WHO classification for endocrine and NET as well as the 2020 WHO classification of neoplasms of the female genital tract [20, 21]. If available, archived histopathologic material was used to create high‐density tissue microarrays (TMAs) as previously described [22]. In short, three 1.0‐mm‐sized cores were punched from representative areas of each patient's tumor out of formalin‐fixed paraffin‐embedded (FFPE) tissue blocks and collected into TMA blocks. The automated Ventana Benchmark Ultra (Ventana Medical Systems, Oro Valley, AZ, USA) was used to immunohistochemically stain 4‐μm‐thick sections of the TMA or FFPE blocks for Ki‐67, SSTR2a, CDX2, PAX8, TTF1, SATB2, ISLET1, OTP, PDX1, and ARX. The choice of immunohistochemistry was based on the profile that is advised for NET of unknown origin (CDX2, PAX8, TTF1, and SATB2) [23], and was augmented with pancreatic (ISLET1, PDX1, and ARX) and lung (OTP) markers, which are frequently used in a research setting. Deparaffinization was achieved with the use of EZ‐prep. A 3.0% H_2_O_2_ solution was then used to block endogenous peroxidase activity, followed by antigen retrieval by boiling in EDTA solution (pH 9, CC1, Roche, Basel, Switzerland) for 24 min. Immunolabeling for Ki67 (Roche: rabbit, clone 30‐9, RTU), SSTR2a (BioTrend, Cologne, Germany: rabbit, polyclonal, 1:25), CDX2 (Cellmarque, Rocklin, CA, USA: EPR2764Y, rabbit, monoclonal, RTU), PAX8 (Abcam, Cambridge, United Kingdom: SP348, rabbit, monoclonal, clone ab227707, 1:50), TTF1 (Ventana Medical Systems: SP141, rabbit, RTU), SATB2 (Cellmarque: EP281, rabbit, monoclonal, RTU), PDX1 (Abcam: GR300167‐8, rabbit, clone EPR3358 [2], 1:50), ARX (Milllipore, Burlington, MA, USA: 3385841, mouse, clone 11F6,2, 1:50), ISLET1 (Cellmarque: 80859, rabbit, clone EP283, 1:100), and OTP (Novus Biologicals, Toronto, Canada: MAB‐03602, mouse, CL11225, 1:200) was subsequently performed with the OptiView DAB IHC detection Kit (Ventana Medical Systems). Assessment of these immunohistochemical stains was done blinded to any patient data, including tumor origin. For these stains, “positive” expression was defined as a histoscore of ≥150 in NET cells, as previously described [24].

### DNA extraction and methylation processing

DNA methylation profiling was performed on a selection of NET no older than 2010 to ensure DNA quality. DNA extraction from representative FFPE tissue sections, bisulfite conversion, and array processing were performed at the core facility of the UMCU, as previously described [17]. DNA methylation analysis was carried out using Infinium MethylationEPICv1 BeadChip (Illumina, San Diego, CA, USA) in accordance with the protocols supplied by the manufacturer. Raw data were exported as IDAT files for further processing. For data processing, the software package *minfi* was used for importing the IDAT files and the *preprocessNoob* function data normalization [25, 26]. Probes were removed if they (1) were located on sex chromosomes, (2) contained single‐nucleotide polymorphisms in the CpG or single‐base extension site, or (3) were found to exhibit cross‐reactivity [27].

### Statistics and data processing

Data are presented as median and interquartile range (IQR) for continuous data and frequency and percentage of cases for categorical data. A chi‐square or Fisher's exact test was used for categorical data and a Wilcoxon rank‐sum test was utilized for continuous data. OS was analyzed with the Kaplan–Meier method and the log‐rank test was used to compare differences between groups [28]. A two‐tailed *p* value of <0.05 was considered significant.

All data analyses were conducted in R Studio v4.1 or higher [29], using the *tidyverse* software collection for data wrangling and *ggplot* for visualization. Two unsupervised approaches were employed: dimensionality reduction using Uniform Manifold Approximation and Projection (UMAP), and hierarchical clustering. For computational efficiency, these analyses were performed on a reduced dataset filtered for the *n* = 5,000 most variable CpG sites (as ranked by variance). UMAP was performed using the *umap* package version 0.2.10.0 with n_neighbours = 15 and min_dist = 0.1 [30]. Unsupervised hierarchical clustering and heatmap generation were obtained using the *ComplexHeatmap* R package version 2.18.0 by Ward's linkage and Euclidean distance. For copy number analysis, raw methylation array data was used. Individual genome‐wide copy number variation plots were generated using the *conumee* R package version 1.36.0 [31] and visually inspected for large‐scale chromosomal aberrations defined as deviations of more than 0.4.

## Results

### Study population

**Figure 1:**
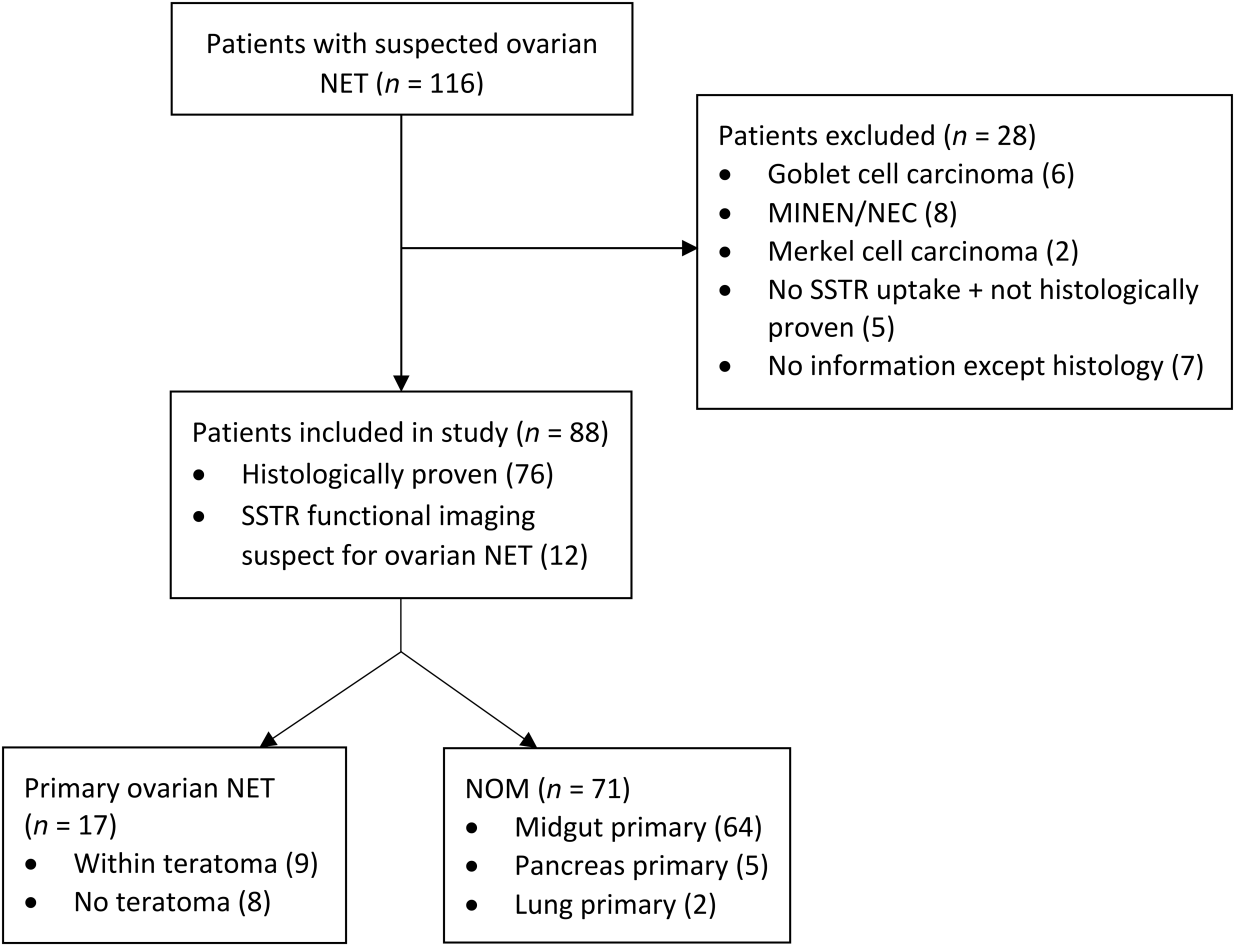
Flow diagram of patient selection. MINEN, mixed neuroendocrine nonneuroendocrine neoplasm; NEC, neuroendocrine carcinoma; NET, neuroendocrine tumor; NOM, neuroendocrine ovarian metastasis; SSTR, somatostatin receptor.

Our search identified 116 patients with ovarian lesions suspected of being a NET. Sixteen of these cases were excluded as pathological review showed them to be either a goblet cell carcinoma metastasis (*n* = 6), an MiNEN/NEC (*N* = 8) or a metastasis of a Merkel cell carcinoma (*n* = 2). An additional 12 cases were excluded because these ovarian NET were not histopathologically proven and did not have any uptake on SSTR functional imaging (*n* = 5), or due to a lack of clinical information (*n* = 7). Thus, 88 patients met our inclusion criteria (Figure 1). For 71 of these patients, a primary tumor was found (64 midgut, 5 pancreatic, and 2 lung NET) and their ovarian lesions were considered to be metastases. For the other 17 patients, a primary tumor was not detected after a thorough review of available computed tomography (CT) and SSTR functional imaging. Hence, these were considered to be PONs. Nine of these PON were found to be combined with a teratoma (PON‐T+), while this was not observed for any of the NOM (Figure 1).

### Clinical characteristics

At the time of ovarian NET diagnosis, patients with PON‐T+ were younger than patients with NOM (*p* = 0.014, median age of 54 versus 62 years), while those with PON‐T− had a similar age to patients with NOM. Patients with NOM more frequently had bilateral ovarian involvement compared with patients with PON (77.5% versus 0%, *p* < 0.001) (Table 1). Extraovarian metastases at ovarian NET diagnosis were present in 67 patients with NOM, in 1 patient with PON‐T−, and none of the patients with PON‐T+. After a median (IQR) follow‐up of 5.0 (2.8; 9.2) years, extraovarian metastatic sites were detected in 70 patients with NOM, 3 patients with PON‐T−, and 1 patient with PON‐T+. Notably, 64 patients with NOM had either peritoneal, mesenteric, or omental metastases at the end of follow‐up, whereas patients with PON in our cohort exclusively developed metastases in the liver (two PON‐T− and one PON‐T+ patient), distant lymph nodes (one PON‐T− patient), bones (two PON‐T− and one PON‐T+ patient) and lungs (one PON‐T− patient). During follow‐up, 31 patients with NOM, 1 patient with PON‐T−, and 1 patient with PON‐T+ died. OS of patients with NOM was shorter than that of patients with PON [median: 8.1 years (IQR: 7.2–12.8) versus. 16.1 years (IQR: 8.6‐not reached), *p* = 0.037], with 5‐year survival rates of 65% and 100%, respectively. OS of patients with PON‐T− did not differ from patients with PON‐T+ (median OS 16 years versus not reached, *p* = 0.32).

**Table 1:** Clinical characteristics

Variable	NOM	PON‐T−	PON‐T+
Total	71	8	9
Age at diagnosis	62 (55–72.5)	61.5 (55–66.8)	54 (43–60)*,†
Year of diagnosis	2011 (2006–2018)	2012 (2005–2018)	2016 (2013–2020)
Lateralization			
Bilateral	55 (77.5)	0 (0)*	0 (0)*
Right	12 (16.9)	2 (25)	7 (77.8)*
Left	4 (5.6)	6 (75)*	2 (22.2)
Underwent oophorectomy			
All patients	55 (77.5)	8 (100)	9 (100)
Bilateral oophorectomy	45 (81.8)	5 (83.3)	6 (66.7)

Values given as *n* (%) or median (IQR).

NOM, neuroendocrine ovarian metastasis; PON‐T−, primary ovarian neuroendocrine tumors without teratomatous components; PON‐T+, primary ovarian neuroendocrine tumors within a teratoma.

* Significant compared to NOM.

† Significant compared to PON‐T−.

CS was present in 46 patients with NOM, in 4 with PON‐T−, and in 1 with PON‐T+. Extraovarian metastases were present in all of the CS patients with NOM and in one PONT‐T− patient. Of the patients with CS who underwent surgical removal of the ovaries without simultaneous tumor debulking and had known CS symptoms or urinary 5‐HIAA measurements before and after oophorectomy, 100% of patients with PON had biochemical (*n* = 2) and symptomatic (*n* = 4) response of their CS. These responses were observed in 36% (*n* = 5) and 27% (*n* = 3) of patients with NOM, respectively.

### Histopathology

**Figure 2:**
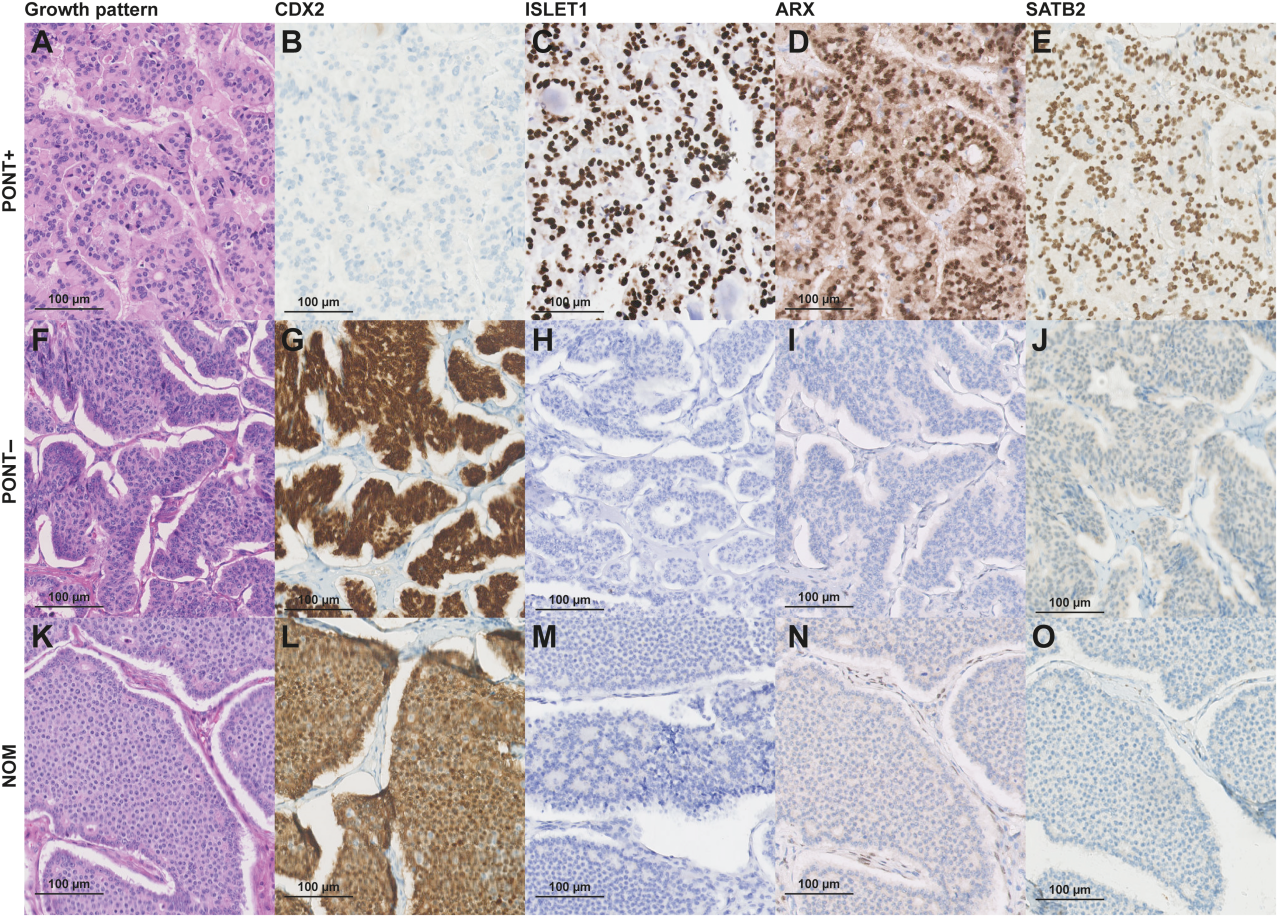
Panel of immunohistochemical characteristics that discriminated best between PON and NOM. NOM, neuroendocrine ovarian metastasis; PON‐T−, primary ovarian NET without teratomatous components; PON‐T+, primary ovarian NET within a teratoma.

Basic histopathological assessment revealed that the Ki67 index of NOM did not differ from PON [median (IQR) of 1% (0–2) for both groups, *p* = 0.34]. However, the mitotic count of NOM was higher than that of PON‐T+ [median (IQR) of 0 (0–1.8) versus 0 (0–0), *p* = 0.042]. NOM and PON‐T− more frequently had an insular growth pattern than PON‐T+ (*p* < 0.001 and *p* = 0.003, respectively). PON‐T− were generally larger than NOM and PON‐T+ [median diameter of 115 versus 50 mm (*p* = 0.001) and 52 mm (*p* = 0.014), respectively] (Table 2). While CDX2 positivity and ISLET1, ARX, and SATB2 negativity were more frequent in NOM compared with PON‐T+ (*p* < 0.01 for all comparisons), no immunohistochemical stains or profile of stains showed differences between NOM and PON‐T− (*p* > 0.05 for all stains, Table 3, Figure 2, and supplementary material, Figure S1). When PON subgroups were compared with each other, PON‐T+ more often showed ARX and ISLET1 expression and less often CDX2 expression than PON‐T− (*p* = 0.039, 0.066, and 0.089, respectively).

**Table 2:** Histopathological results in ovarian NET

Variable	NOM	PON‐T−	PON‐T+
Total	54	8	9
Ki‐67 index (% positive cells)	1 (0–2)	1 (0–2)	1 (0–2)
Mitotic count (/mm^2^)	0 (0–1.8)	0 (0–1)	0 (0–0) *
Morphology			
Insular	50 (92.6)	7 (87.5)	1 (11.1)*,†
Trabecular	2 (3.7)	1 (12.5)	1 (11.1)
Insular and trabecular	0 (0)	0 (0)	2 (22.2)*
Strumal	1 (1.9)	0 (0)	4 (44.44)
Diffuse	0 (0)	0 (0)	1 (11.1)
Solid	1 (1.9)	0 (0)	0 (0)
Size‡ (mm)	50 (28–72.5)	115 (70–170)*	52 (10–60)†

Values given as *n* (%) or median (IQR).

NOM, neuroendocrine ovarian metastasis; PON‐T−, primary ovarian NET without teratomous components; PON‐T+, primary ovarian NET within a teratoma.

* Significant compared to NOM.

† Significant compared to PON‐T−.

‡ Diameter of ovarian NET, mean if bilateral.

**Table 3:** Immunohistochemistry results

Marker (tissues stained per subgroup)	NOM	PON‐T−	PON‐T+
Total	54	8	9
SSTR2a (47/8/8)	38 (80.9)	5 (62.5)	5 (62.5)
CDX2 (43/8/8)	37 (86)	6 (75)	2 (25)*
PAX8 (43/7/6)	0 (0)	0 (0)	1 (16.7)
ISLET1 (39/5/5)	3 (7.7)	0 (0)	4 (80)*,†
ARX (39/6/5)	2 (5.1)	1 (16.7)	4 (80)*
PDX1 (40/6/5)	7 (17.5)	0 (0)	1 (20)
TTF1 (43/8/8)	1 (2.3)	0 (0)	1 (12.5)
SATB2 (43/8/7)	1 (2.4)	2 (25)	4 (57.1)*

Values given as *n* (%) of positive stains, defined as histoscore ≥150.

NOM, neuroendocrine ovarian metastasis, PON‐T−, primary ovarian neuroendocrine tumors without teratomatous components, PON‐T+, primary ovarian neuroendocrine tumors within a teratoma.

* Significant compared to NOM.

† Significant compared to PON‐T−.

### DNA methylation profile analysis

**Figure 3:**
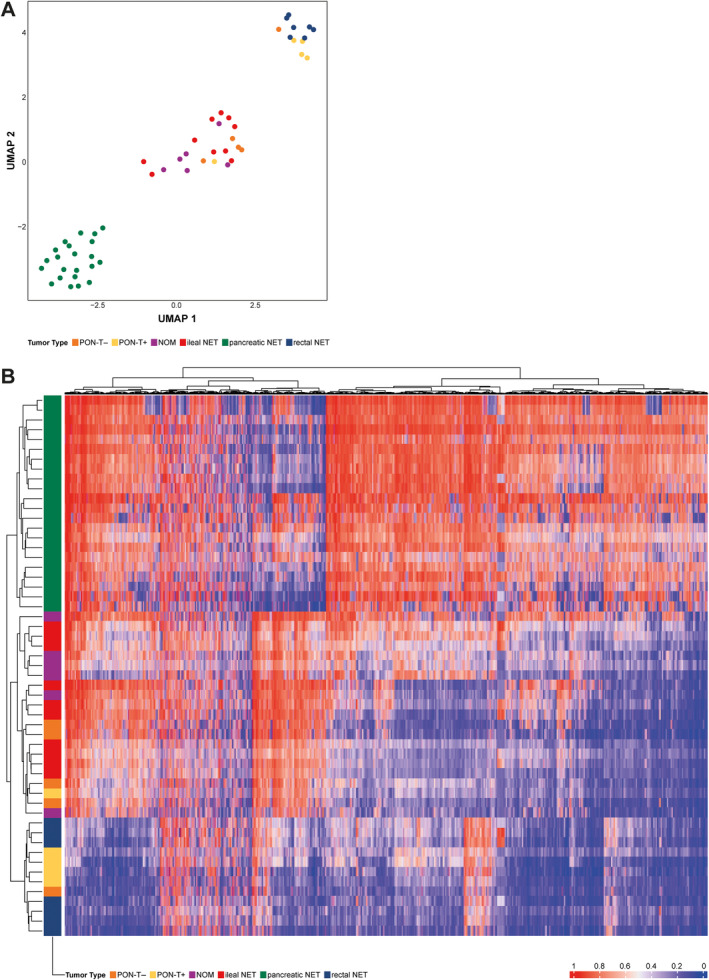
Two unsupervised analyses, including dimensionality reduction using (A) Uniform Manifold Approximation and Projection (UMAP) and (B) hierarchical clustering, both identifying three clusters: (1) a cluster of pancreatic NET; (2) a cluster of rectal NET and primary ovarian neuroendocrine tumors within a teratoma (PON‐T+), and (3) a cluster of ileal NET with primary ovarian neuroendocrine tumors (PON‐T−) and neuroendocrine ovarian metastases (NOM).

DNA methylation analysis of NET tissue was performed in 5 PON‐T+, 5 PON‐T−, 6 NOM of midgut origin, 10 ileal NET, 22 pancreatic NET, and 7 rectal NET. Unsupervised analysis revealed three clusters using UMAP (Figure 3A). The first cluster contained all pancreatic NET, which were effectively differentiated from the other primary origins studied. The second cluster consisted of rectal NET and PON‐T+, with one single PON‐T−. This case displayed a trabecular growth pattern with negative CDX2 and positive ARX staining and no chromosome 18 loss (supplementary material, Figure S1B–D,G). The final cluster contained ileal NET together with PON‐T− and NOM. This cluster included a single case of PON‐T+, which showed chromosome 18 loss and negative staining of CDX2, ARX, and ISLET1 (supplementary material, Figure S1B,D,G,H). Moreover, this PON‐T+ patient was the only one in which CS was present. Review of the teratomatous components of this PON‐T+ showed that it was the only one with respiratory and central nervous system tissue next to skin tissue. Small intestinal tissue was not seen in this case, whereas the other PON‐T+ cases featured only skin and/or thyroid tissue. Unsupervised hierarchical clustering showed corresponding clustering into three groups (Figure 3B). Chromosome 18 loss was found in 4/6 (67%) NOM, 2/5 (40%) PON‐T−, 1/5 (20%) PON‐T+, 5/10 (50%) ileal NET, and 1/10 (10%) rectal NET (supplementary material, Figure S1B).

## Discussion

This manuscript set out to discriminate NOM from PON with and without teratomatous components. In our study, all PON were unilateral and 53% of them were found within a teratoma. In contrast, 78% of NOM were bilateral and none were associated with a teratoma. The median diameter of PON‐T− was larger than NOM and PON‐T+, and patients with PON‐T+ were younger at diagnosis than those with NOM or PON‐T−. Patients with PON‐T− more frequently developed metastases than those with PON‐T+ and none developed peritoneal, mesenteric, or omental metastases, while this was the case for the majority of patients with NOM. Even though there were histopathological and epigenetic differences between NOM and PON‐T+, NOM and PON‐T− displayed similar immunohistochemistry, growth patterns, DNA methylation profiles, and loss of chromosome 18.

In line with previous results, this study showed that finding an ovarian NET within a teratoma is the strongest indicator for a primary ovarian origin [8, 14]. Minimizing the possibility of a nonovarian origin is an important first step in the management of a patient with a suspected PON. The presence of a nonovarian primary NET indicates stage IV disease, which warrants a more aggressive treatment and follow‐up strategy. This is supported by our results and those of others, which show that patients with PON have a substantially longer OS when compared to patients with NOM [14]. A study of 329 patients with PON showed that OS is higher and the rate of developing metastases is lower in patients with PON‐T+ when compared with PON‐T− patients [4]. Our results, even though based on a smaller number of patients, showed a similar trend. Only one PON‐T+ patient died in our cohort, shortly after developing hepatic and bone metastases with a lesion in the pancreas 9 years after curative oophorectomy, suggesting a secondary primary tumor instead of recurrence of disease. Therefore, when dealing with a suspected PON, it is paramount to perform a complete oophorectomy and to sample as much material of the ovary for histopathological assessment as possible in order to minimize the chance of missing a small teratomatous component. Importantly, ISLET1, ARX, and CDX2 seem to be the most discriminative markers to differentiate between PON‐T− and PON‐T+.

When an ovarian NET is found without the presence of a teratoma and without evidence of a primary lesion elsewhere, it can be very difficult to differentiate between a PON and a NOM. This study showed that PON‐T− have similar growth patterns, immunohistochemistry, and DNA methylation profiles as NOM. Additionally, PON‐T− exhibited chromosome 18 loss in 40% of cases, a characteristic frequently observed in ileal NET [32]. Hence, it is possible that PON‐T− are, in fact, solitary metastatic lesions of an unfound primary midgut NET, and a similar follow‐up strategy may be justified for these patients. This hypothesis is contradicted by the finding that none of the three PON‐T− that developed metastases showed peritoneal, mesenteric, or omental metastases, while 90% of the NOM developed these metastases. We postulate that this is due to our small sample size, as peritoneal metastases have been described in patients with PON‐T− [4]. Following these observations, a thorough review of imaging and, if operated on, intraoperative manual inspection of the small intestine should be performed for patients with PON‐T− to identify a possible unfound midgut NET.

The assumption that a PON‐T− is a solitary metastatic lesion of an unfound primary midgut NET could also explain the development of a PON outside of the context of a teratoma. Other hypotheses include the possibility of the NET being the sole component of a teratoma present in the ovary, or that there are teratomatous components present, which are either missed or not submitted for histopathological assessment. However, due to the observed histopathological and molecular differences between PON‐T+ and PON‐T− both in our study and in other studies [4], it is unlikely that this is the case for the majority of PON‐T−. Alternatively, there could be neuroendocrine cells in nonneoplastic ovaries that develop into an NET, as is the case for NET of the prostate [33]. While this has been described in a study published in 1982 that utilized an argentaffin stain [34], we did not encounter neuroendocrine cells when we stained two normal ovaries with chromogranin A and synaptophysin (data not shown).

One interesting finding is that there were two cases in the epigenetic analysis that clustered differently from their subgroup: one PON‐T− that clustered with PON‐T+ and vice versa. As the PON‐T+ aligning with the cluster of PON‐T−, ileal NET and NOM also showed chromosome 18 loss, we postulated the presence of ileal tissue within the teratomous component. Review of the teratomatous components of this PON‐T+ case showed no small intestinal tissue in the particular case. However, the presence of ileal tissue cannot be excluded as we observed a greater variety of tissue types in the teratoma of this case compared to the other PON‐T+. Likewise, for the PON‐T− clustering with the PON‐T+ and rectal NET, it could be possible that teratomatous components were not included in the sections, causing this case to be incorrectly classified as PON‐T−. Alternatively, the PON‐T− might, in fact, be an ovarian metastasis of a rectum NET and the PON‐T+ a midgut NET metastasized into a teratoma, for which the primary tumor was not visible on imaging after 12 years of follow‐up. Another remarkable finding was that most PON‐T+ seemed to have similarities with rectal NET, based on both DNA methylation and immunohistochemical profile. As colon tissue was not found in the teratoma component of these cases, the cause of this molecular clustering remains obscure, but could suggest a common lineage.

Our study showed that CS is frequently present in patients with ovarian NET. While other reports have suggested a similar prevalence, they based their definition of CS on either symptoms of diarrhea or flushing or elevated 5‐HIAAs [4, 7, 8, 9]. This is the first study to report CS incidence in this patient group according to the most recent definition [6]. Our study and the work of Robboy *et al* suggest that oophorectomy can be a good treatment option to reduce CS symptoms as well as to reduce 5‐HIAA levels [8]. It should be noted that the disease of patients with PON was generally confined to the ovaries, explaining the response rate of 100% for these patients. For patients with NOM, however, oophorectomy can be considered in cases with extensive ovarian tumor bulk, perhaps combined with other tumor debulking like liver‐directed therapy, for treatment‐refractory CS patients with NOM.

An inherent limitation in studies regarding specific subgroups of NET patients is their small sample size, which increases the possibility of type II statistical errors. This is, however, the largest cohort of patients with NOM described thus far and the largest cohort of patients with PON for which a central pathology review was performed. As shown by the 16 patients with suspected ovarian NET that ended up having a different diagnosis (Figure 1), central pathology review is essential to conduct retrospective cohort studies. It is also paramount in order to minimize the possibility of a missed teratoma.

## Conclusions

The presence of a teratoma and lateralization performed best in differentiating between PON and NOM. PON‐T− displayed similar immunohistochemistry, growth patterns, DNA methylation profiles, and loss of chromosome 18 as NOM of midgut origin. Therefore, when dealing with patients with an ovarian NET localization, a unilateral NET within a teratoma should be treated as a PON. When a teratomatous component is not present, the molecular signature suggests that the ovarian NET is likely of midgut origin. For these patients, thorough review of imaging should be performed to identify a possible undetected midgut NET and a corresponding follow‐up strategy may be recommended.

## Author contributions statement

MCFM collected data from electronic patient records, carried out the formal data analysis and wrote the original draft of the manuscript. AVDV and CG performed the DNA methylation analysis. M‐LFV performed the central pathology review. MCFM, JH, WWH and QGLS thoroughly reviewed the PON cases to minimize the possibility of a missed primary origin. MCFM carried out the conceptualization of the study and AVDV, LAAB, WWH, M‐LFV and JH participated in the study conceptualization. All authors reviewed the results, critically reviewed and edited the manuscript and approved the final version of the manuscript.

## Supporting information


**Figure S1.** Chromosome 18 loss, growth pattern, and immunohistochemistry results projected over UMAP

## Data Availability

DNA methylation data are available upon reasonable request. Code used for the generation of results and figures can be accessed at https://github.com/averschuur/Ovary.
