# Supplementary material for: Clinicopathological and epigenetic differences between primary neuroendocrine tumors and neuroendocrine metastases in the ovary
Source: J Pathol Clin Res. 2024 Nov 8;10(6):e70000. doi: 10.1002/2056-4538.70000 (PMC11544441; doi:10.1002/2056-4538.70000)
Supplement: Supplementary file 1 — Figure S1. Chromosome 18 loss, growth pattern, and immunohistochemistry results projected over UMAP [file CJP2-10-e70000-s001.pdf]

Clinicopathological and epigenetic differences between primary neuroendocrine tumors and neuroendocrine metastases in the ovary

MCF Mulders *et al. J Pathol Clin Res* https://doi.org/10.1002/2056-4538.70000

Supplementary Figure S1

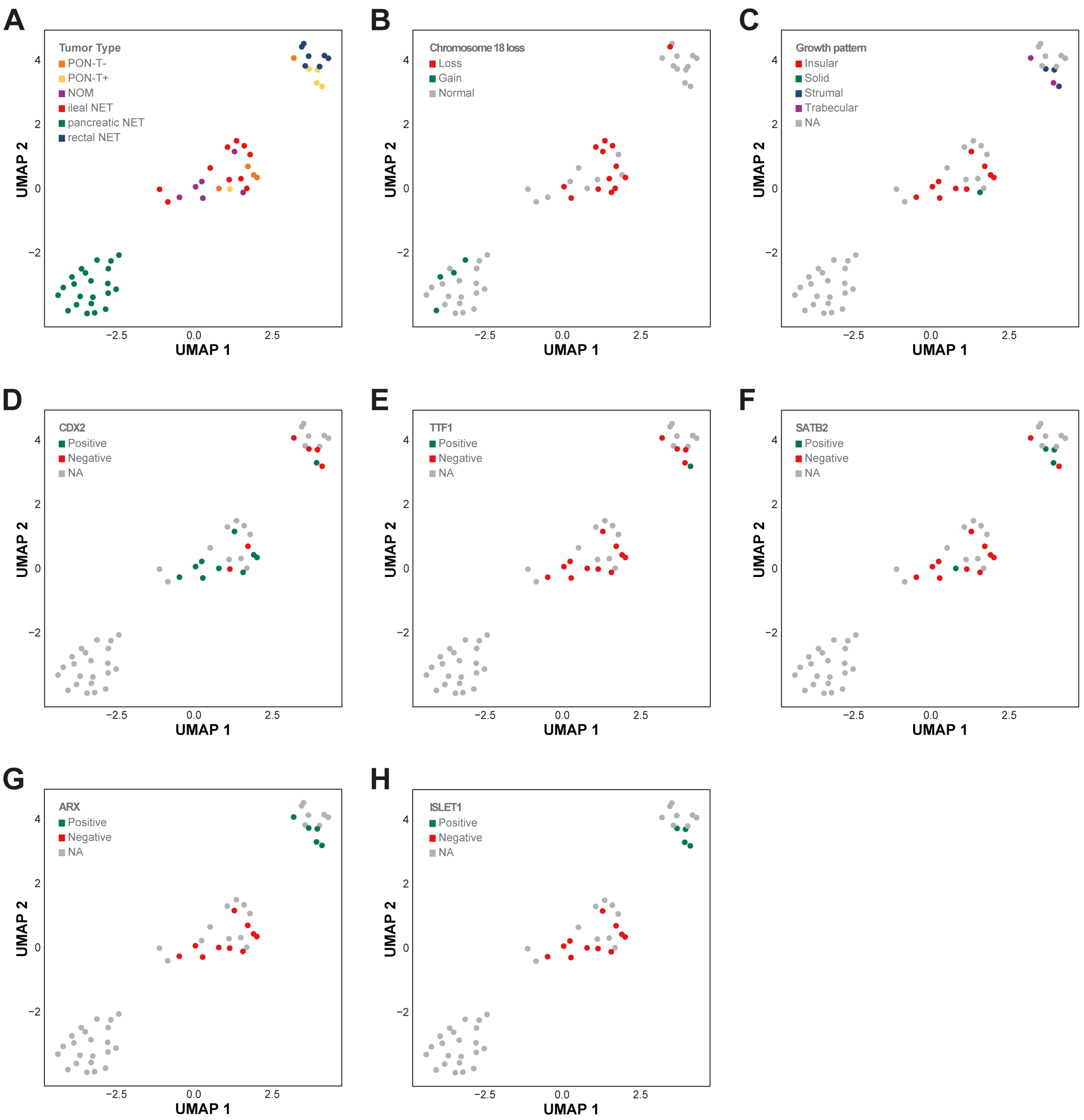

Figure S1. Chromosome 18 loss, growth pattern and immunohistochemistry results projected over Uniform Manifold Approximation and Projection (UMAP). The reference UMAP in (**A**) illustrates various tumor types. NET = neuroendocrine tumor, NOM = neuroendocrine ovarian metastasis, PON-T- = primary ovarian neuroendocrine tumors with no teratomous components, PON-T+ = primary ovarian neuroendocrine tumors within a teratoma. Subsequent panels show specific features: (**B**) Chromosome 18 loss, (**C**) Growth pattern, (**D**) CDX2 immunohistochemistry, (**E**) TTF1 immunohistochemistry, (F) SATB2 immunohistochemistry, (**G**) ARX immunohistochemistry, and (**H**) ISLET1 immunohistochemistry.
